# Dysfunctional customer behavior influences on employees’ emotional labor: The moderating roles of customer orientation and perceived organizational support

**DOI:** 10.3389/fpsyg.2022.966845

**Published:** 2022-10-10

**Authors:** Pengfei Cheng, Jingxuan Jiang, Sanbin Xie, Zhuangzi Liu

**Affiliations:** School of Economics and Management, Xi'an University of Technology, Xi'an, China

**Keywords:** dysfunctional customer behavior, perceived organizational support, customer orientation, conservation of resources theory, emotional labor

## Abstract

Despite increasing interest being given to dysfunctional customer behavior in multiple service sectors, it is unclear how and why different types of dysfunctional customer behavior (verbal abuse, disproportionate demand, and illegitimate complaint) affect frontline employees’ emotional labor during the service interactions. Drawing upon the conservation of resources theory, we propose a conceptual model in which verbal abuse, disproportionate demand, and illegitimate complaint differentially influence frontline employees’ emotional labor strategies (surface acting and deep acting). Further, the boundary conditions of these relationships are considered by introducing perceived organizational support and customer orientation as moderators. Using survey data from 436 frontline employees of five call centers in China, hypotheses were tested through a hierarchical regression analysis. The results indicated that verbal abuse and illegitimate complaint exerted positive effects on surface acting. Particularly, these positive effects were weaker when frontline employees perceived organizational support was high. Also, verbal abuse’s positive effect on surface acting was weaker when frontline employees’ customer orientation was high. Customer’s verbal abuse, disproportionate demand, and illegitimate complaint negatively influenced frontline employees’ deep acting. The negative effect of disproportionate demand on deep acting was weaker when perceived organizational support was high. However, when frontline employees’ customer orientation was high, the negative effects of disproportionate demand and illegitimate complaints on deep acting were weaker.

## Introduction

To survive within the fiercer competition environments, service firms are increasingly realizing the importance of service experience. Given frontline employees’ pivotal role in creating a good service experience, they are expected to display positive emotions during the service interaction (Hur et al., 2022). Since the Seminal work of Hochschild (1983), emotional labor has been a construct of increasing interest for both academics and practitioners because of its relationship with service outcomes and employee wellbeing. Hochschild (1983) defined emotional labor as how employees regulate and display their emotions during a service encounter, and described two types of emotional labor strategies: surface acting and deep acting. Specifically, in surface acting, employees only change their outward emotional display without genuinely altering how they feel (Hochschild, 1983). In contrast, deep acting is described as a more sincere act (Grandey, 2003), in which employees attempt to adjust their felt emotions so that a genuine, organizationally desired emotional display can follow (Hochschild, 1983). Prior research on emotional labor suggests that only genuine emotional display (deep acting) of employees contributes to positive outcomes, such as customer emotional experience (Liu et al., 2019) and perceived service quality (Gong et al., 2020). However, surface acting causes negative outcomes such as customer negative emotion (Ashtar et al., 2021), cognitive weariness (Sousan et al., 2022), and burnout (Kim, 2020).

Given the important role emotional labor plays in shaping service experience (Gong et al., 2020), considerable research focuses on identifying antecedents to emotional labor. A stream of research investigates the effects of organizational factors on emotional labor, such as leadership (Lu et al., 2019), organizational justice (Shapoval, 2019), corporate social responsibility (Oh et al., 2019; Shin and Hur, 2020), and organizational dehumanization (Nguyen et al., 2022). Another stream of research explores antecedents of emotional labor from the employee perspective, such as dispositional traits (Lee and Madera, 2019), motivation (Hur et al., 2022), and demographic (Rasheed-Karim, 2020). Due to the interactive nature of service encounters, an emerging trend in the emotional labor literature has been shifting interest to regarding how customers in a service encounter influence employees’ emotional labor (Szczygiel and Bazińska, 2021). Medler-Liraz (2016) found that customer displays positive emotion during the interaction decrease employees’ need for engaging in deep acting. The evidence from Yoo (2016) suggests that customer participation exhibits the predicted negative influence on surface acting as well as the positive effect on deep acting. However, the evidence from Choi and Lawry (2020) suggests that customer participation positively influences employees’ surface acting and, in turn, job stress. These studies have dominantly focused on customer positive behaviors’ effects on employee’s emotional labor and have ignored customer negative behaviors’ effects. In addition, the mixed results of the relationship between customer behaviors and emotional labor imply the boundary condition should be further explored.

During the service interaction, dysfunctional customer behavior is endemic and prevalent in many service sectors, especially in call center, airline, and hospitality industries (Boukisa et al., 2020). It brings new challenges for employees and organizations (Bani-Melhem et al., 2020). Harris and Reynolds (2003) defined dysfunctional customer behavior (DCB) as customer behavior thoughtless or abusive that causes problems for the service organization, its employees, and/or other customers. Recently, Kang and Gong (2019) redefined DCB as customer actions in service settings that deliberately violate the generally accepted norms of conduct for how to treat employees, which focuses on customer actions targeting employees. In line with Kang and Gong (2019), we conceptualize DCB as consisting of three dimensions: verbal abuse, disproportionate demand, and illegitimate complaint. Exposure to DCB may lead to several negative consequences for employees among which are employee withdrawal (Kang and Gong, 2019) and turnover (Gong and Wang, 2019). Although these negative consequences of DCB are closely related to emotional labor, few studies empirically examine the relationship between DCB and emotional labor.

To address these research gaps and respond to the call of Harris and Reynolds (2003) for examining the consequences of DCB more holistically, we aim to explore the relationship between DCB and employees’ emotional labor. As a widely cited theory to explain why employees take different emotion regulation strategies (deep acting and surface acting), conservation of resources (COR) theory suggests that people strive to obtain, retain, protect, and foster valued resources and minimize any threats of resource loss. Threats to resource loss are usually in the form of job demands (e.g., DCB) and the energy and efforts expended toward meeting such demands. Drawing upon the COR theory, we propose a model in which three dimensions of DCB influence frontline employees’ emotional labor strategies. Furthermore, by introducing perceived organizational support (POS) and customer orientation, which represent external and internal resources, as moderators, the boundary conditions of the relationship between DCB and emotional labor are considered. The findings may enrich the knowledge of the consequences of DCB and provide effective managerial tools for managers to buffer the negative influence of DCB on frontline employees’ emotional labor.

## Theoretical background and hypothesis development

### Dysfunctional customer behavior

Frontline employees in service sectors frequently suffer from customers’ deliberate violation of service interaction norms (Arvan et al., 2019; Kang and Gong, 2019). Scholars use the term dysfunctional customer behavior to define customer actions in service encounters that deliberately violate the generally accepted norms of conduct for how to treat employees (Reynolds and Harris, 2009; Kang and Gong, 2019). Other similar items were used interchangeably by previous research include deviant customer behavior (Harris and Daunt, 2011), customer misbehavior (Fullerton and Punj, 2004), customer mistreatment (Park and Kim, 2019), and problem customers (Bitner et al., 1994). Despite acknowledging DCB is a multidimensional concept, most prior research focuses signal specific form of DCB, such as verbal abuse (Cho et al., 2020), and illegitimate complaint (Kim and Baker, 2019; Arora and Chakraborty, 2020). In recent years, Kang and Gong (2019) identified three types of DCB that target frontline employees in service settings, namely verbal abuse, disproportionate demand, and illegitimate complaint. Verbal abuse refers to customers’ impolite verbal communication with employees such as sarcasm, yelling, and swearing; Disproportionate demand is defined as the excessive demands by customers that may be impossibly contented through employees (Kang and Gong, 2019). Furthermore, customers may use illegitimate complaints as a negotiation to gain greatest redress and attention from service providers (Kang and Gong, 2019). Given the purposes of this study, consistent with Kang and Gong (2019), we focus on the employee-target DCB: verbal abuse, disproportionate demand, and illegitimate complaint.

With the proliferation of customer service positions, DCB is becoming more prevalent in different service scenarios (Bani-Melhem et al., 2020), and causes great damage to frontline employees. In a meta-analysis, Arvan et al. (2019) reported that customer mistreatment leads to both employees’ psychological strains and behavior strains. According to the emotional contagion theory, DCB causes employees to experience negative emotions such as frustration and irritation during service interactions (Hu et al., 2018). For example, Chi et al. (2018) reported that DCB is associated with frontline employees’ negative emotions which even can spill over into their home lives, subsequently creating work–family conflicts. Furthermore, frontline employees may have to regulate negative emotions (emotional labor) when they are confronted with DCB, which may lead them to feel emotional exhaustion. The relationship between DCB and employee emotional exhaustion has been addressed by many scholars. Kim et al. (2018) suggest that verbal abuse of customers is positively related to employees’ emotional exhaustion. Szczygiel and Bazińska (2021) further state that customer incivility’s effect on emotional exhaustion is mediated by surface acting and moderated by emotional intelligence. In addition, customer incivility’s also is a predictor of employees’ behavior such as retaliatory behaviors (Gong and Wang, 2019), sabotage service (Hwang et al., 2021), and turnover (Bamfo et al., 2018). However, the negative factors of customers (e.g., customer verbal abuse, illegitimate complaint, disproportionate demand) are generally believed to be difficult to manage by the service firms. Thus, improving employees’ coping abilities possibly might be a key method, while little attention has been paid to exploring DCB’s role in influencing employees’ positive behavior such as emotional labor (Szczygiel and Bazińska, 2021).

### Conservation of resources theory

Conservation of resources (COR) theory suggests that individuals endeavor to possess, obtain, retain, and protect valuable resources that may include socio-emotional support, conditions, and emotional energy (Hobfoll, 2001). The resources that are possessed by individuals are dynamic. During the service encounters, frontline employees may perceive a constant depletion of their resources, while they also attempt to maintain the maximization of valuable resources through gaining external resources (Hobfoll et al., 2018). However, given the importance of individual resources, employees are prone to adopt strategies at work to avoid or reduce resource depletion, motivated by resource protection (Hobfoll, 2001).

**Figure 1:**
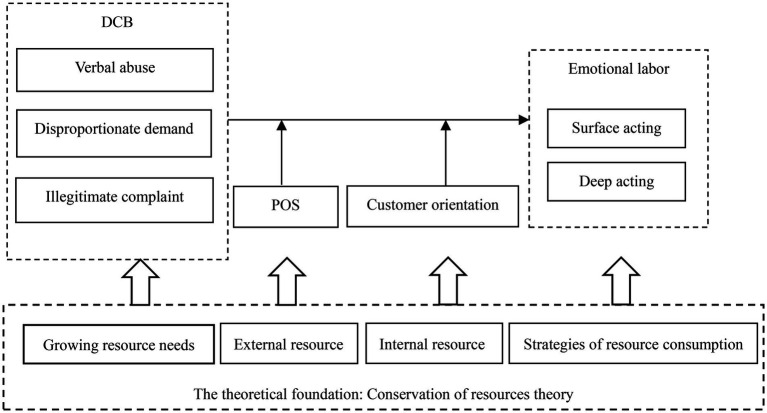
Conceptual model.

Frontline employees frequently perform emotional labor to obey organizational emotional display rules, which will deplete their valuable resources such as emotional energy and patience (Chi and Grandey, 2019). However, two emotional labor strategies (surface acting versus deep acting) result in a different extent of resource depletion for employees. When employees engage in deep acting, they invest more effort in altering their inner feelings (Morris and Feldman, 1996; Grandey et al., 2012), in turn, consume more individual resources. In contrast, when employees use surface acting, they deplete fewer resources. Thus, surface acting is a strategy for employees to “conserve” resources. Employees need to deplete their valuable resources in responding to each customer’s needs, especially meeting non-routine work demands such as DCB (Okan et al., 2021). Therefore, drawing on COR theory, we propose that employees are motivated to retain and protect valuable resources and use corresponding emotional labor strategies when they are faced with DCB (see Figure 1). In addition, employees’ behavioral strategies are also related to their access to an effective complement of resources. Thus, we propose that POS and customer orientation, as important constructs reflecting employees’ access to complementary resources, moderate the impact of DCB on employees’ emotional labor. The conceptual model of this research is shown in Figure 1.

### Dysfunctional customer behavior and employees’ emotional labor

#### Verbal abuse and employees’ emotional labor

According to emotional contagion theory, emotions can spread between individuals through presenting both verbal and nonverbal cues (Hatfield et al., 2011; Liu et al., 2019). Actually, verbal behaviors are so import in emotional interaction that Hwee Hoon et al. (2004) even use greeting and thanking which represent verbal behaviors to measure individual’s displaying positive emotions and smiling and eye contact were nonverbal behaviors. Given the importance of verbal behavior during the emotion contagion process, customer’s verbal abuse, as a typical negative emotion expression, will evoke negative emotions in frontline employees. Namely, frontline employees are “infected” by customers’ negative emotions *via* verbal abuse. For frontline employees who are expected to display positive emotions, experiencing negative involves devoting more resources to regulating their emotions. Furthermore, customer verbal abuse may reduce frontline employees’ self-esteem, confidence, and self-efficacy (Sliter and Jones, 2016; Hwang et al., 2021). All of these are fundamental job resources for frontline employees. In addition, verbal abuse, as a major interpersonal stressor, would undermine the customer-employee relationship and, in turn, prevent frontline employees from gaining personal resources.

Taken together, customers’ verbal abuse not only depletes employees’ valuable emotional resources through emotional contagion process but also impedes employees from restoring resources from service interactions. According to the COR theory (Hobfoll et al., 2018), when customers’ verbal abuse strengthens frontline employees’ perception of resource loss threats, they are more likely to engage in surface acting which depletes less resources, rather than deep acting which depletes more resources.

*H1a*: Verbal abuse is positively related to employees’ surface acting;

*H1b*: Verbal abuse is negatively related to employees’ deep acting.

#### Disproportionate demand and employees’ emotional labor

Although service firms expect employees to try their best to meet customer demands, customers’ disproportionate demands (e.g., special treatment) are usually beyond frontline employees’ competence. To meet customers’ disproportionate demand, frontline employees have to take extra workload (Hwang et al., 2021), which implies more physical effort devoting. Customer’s disproportionate demands, as a kind of job demands (Ashtar et al., 2021), according to the job demand-resources (JD-R) theory (Bakker and Demerouti, 2017), will increase frontline employees’ resource depletion. Because they need to devote much more time and effort to ensure their productivity. In addition, customers’ disproportionate demands could ruin frontline employees’ moods and, in turn, result in employees’ negative emotions (Wang et al., 2011; Wu et al., 2019). For service frontline employees who are expected to display positive emotions, experiencing negative emotions will make them invest efforts to regulate their emotions to obey the display rules. According to COR theory (Hobfoll, 2001), when frontline employees are confronted with resources loss caused by customer’s disproportionate demands, they will take surface acting strategy which depletes less personal resources to merely “fake” emotions, rather than deep acting strategy which depletes more personal resources to change inner emotions and express genuine emotions. (Goussinsky and Livne, 2016). Thus, we propose:

*H2a*: Disproportionate demand is positively related to employees’ surface acting;

*H2b*: Disproportionate demand is negatively related to employees’ deep acting.

#### Illegitimate complaint and employees’ emotional labor

Service firms usually place great emphasis on customer complaints (Kang and Gong, 2019). Complaints not only can serve as a communication channel for customers to express their dissatisfaction and protect their self-interest when suffering service failure but also provide valuable opportunities for service firms to take recovery actions and avoid customer switching. However, customers, motivated by opportunism or self-interest, may take advantage of this chance to perform dysfunctional behaviors (e.g., illegitimate complaint) to obtain interests (Harris and Reynolds, 2003). For instance, customers may make illegitimate complaint without an actual experience of service failure (Baker et al., 2012). Illegitimate complaints from customers are so common that they bring enormous challenges to frontline employees and service organizations. On the one hand, illegitimate complaints are likely to be detrimental to employees’ emotions. For example, Huang and Miao (2016) found that illegitimate complaint by consumers is positively related to negative emotions of employees (e.g., frustration and sadness). Frontline employees that felt negative emotions need devote more psychological resources to smoothen the process of service delivery (Huang and Miao, 2016). Thus, we can infer that illegitimate complaints make frontline employees lose more resources during the service encounters.

On the other hand, illegitimate complaint may thwart employees from gaining resources from service encounters. Harmony interactions between frontline employees and customers lead to high service performance which can improve frontline employees’ self-efficacy and sense of personal accomplishment. No matter self-efficacy or sense of personal accomplishment are important psychological resources for employees (Wang et al., 2011). When frontline employees suffer from illegitimate complaints by customers, it is difficult for employees to build customer-employee interpersonal relationships and feel self-efficacy and individual accomplishment. Furthermore, illegitimate complaints can also undermine the mutual trust employees and customers, which will make the service interaction ineffective. Therefore, when employees encountered customers’ illegitimate complaints, they are prone to perceive fatigue and depleted psychological resources (e.g., emotional energy).

According to COR theory, when individuals face threats of resource loss, they strive to retain and protect valuable resources (Hobfoll, 2001). To minimize the loss during the service encounters, frontline employees who are coping with customers’ illegitimate complaints may choose to distance themselves from customers by engaging in surface acting (Grandey et al., 2007). Compared with engaging in surface acting, engaging in deep acting needs frontline employees to devote more cognitive efforts and emotional energies and, in turn, results in more individual resources loss (Morris and Feldman, 1996; Grandey et al., 2012). Taken together, we propose:

*H3a*: Illegitimate complaint is positively related to employees’ surface acting;

*H3b*: Illegitimate complaint is negatively related to employees’ deep acting.

### Moderating effects of perceived organizational support

Eisenberger et al. (1986) defined perceived organizational support (POS) as “employees’ perceptions that the organization values their contributions and cares about their wellbeing.” POS provides employees tangible (material rewards) and/or intangible (emotional energy) resources, and is associated with employees’ abilities and attitudes, such as self-efficacy and job satisfaction (Caesens et al., 2017; Islam and Ahmed, 2018). According to Chan and Wan (2012), POS can be viewed as a kind of resource which could enable employees to cope with stressful job conditions, such as the DCB scenarios. POS can shift employees’ attention away from job stressors and help them reinterpret the job environment from a more optimistic perspective so that it seems less threatening (Yoo and Arnold, 2016; Wen et al., 2019). Actually, in the emotional labor literature, there are two similar strategies (attentional deployment and cognitive reappraisal) that are primarily taken by employees to engage in deep acting. Attentional deployment refers to changing the experience of emotions by shifting one’s attention to specific aspects of the situation (Gross, 2008); Cognitive reappraisal refers to evoking specific emotions by reappraising the situation (Gross, 2008). Hence, we can expect that frontline employees with high POS are prone to take deep acting, rather than surface acting, to deal with DCB.

POS also fulfills employees’ socioemotional needs for emotional support, self-esteem, affiliation, and social approval (Eisenberger et al., 2020), which are bases of individual’s psychological resources. When frontline employees perceive high organizational support, they can obtain personal resources from their organization. High-level personal resources can make employees not hesitate to invest more cognitive efforts in evoking specific emotions and expressing them authentically (deep acting; Hur et al., 2013), rather than only change their outward emotional display without genuinely altering how they actually feel (surface acting). Furthermore, POS make frontline employees feel obligation toward their organization as well as the expectation that their efforts will be rewarded (Thompson et al., 2020). As a result, employees with high POS would be motivated to internalize organization’s goal and value by complying with emotion display rules, such as “service with smile.” Given that deep acting is a kind of discretionary behavior driven by intrinsic motivation (Hur et al., 2022), it is reasonable to infer that frontline employees with high POS are more likely to respond DCB by deep acting. In contrast, employees with low POS are more likely to engage in surface acting to respond DCB. Taken together, we propose:

*H4*: The positive influences of verbal abuse (H4a), disproportionate demand (H4b), and illegitimate complaint (H4c) on employees’ surface acting are weaker (stronger) when POS is high (low).

*H5*: The negative influences of verbal abuse (H5a), disproportionate demand (H5b), and illegitimate complaint (H5c) on employees’ deep acting are weaker (stronger) when POS is high (low).

### Moderating effects of customer orientation

Customer orientation (CO) is defined as an employee’s tendency or predisposition to meet customer needs in an on-the-job context (Brown et al., 2002). Customer orientation can influence frontline employees’ job perception, attitudes, and behaviors in service interaction with customers (Huang and Brown, 2016; Kim and Qu, 2020). Firstly, CO can reduce the extent of job stress employees perceive when confront with DCB. Although DCB interrupts the flow of normal service processes and routines, CO can protect employees from suffering role ambiguity and role conflict. Because CO provides frontline employees with strong guidance regarding the purpose of job (Huang and Brown, 2016). Frontline employees with high CO can cope with DCB more effectively than employees with low CO. Thus, CO can serve as a buffer from the potentially deleterious effects of DCB.

Second, CO increases employees’ work engagement or the level of effort spent in dealing with DCB. According to the COR theory, CO as a personal resource enhances employees’ job engagement and confidence (Zablah et al., 2012), which, in turn, fosters employees to provide an expression of genuine emotion (deep acting) rather than a low level of authenticity of emotional display (Yoo and Arnold, 2014). Also, CO may act as an intrinsic motivator that drives employees to invest their job efforts in satisfying customers’ needs (Zablah et al., 2012; Moon et al., 2019). Hence, when facing DCB, frontline employees with high CO are likely to change their internal feelings and display authentic emotions through a conscious and effortful process. In contrast, frontline employees with low CO believe that they do not have enough resources and methods to cope with DCB no matter how much effort they invest. This will undermine employees’ motivation and thereby consequently leads to engaging in surface acting. Taken together, we propose:

*H6*: The positive influences of verbal abuse (H6a), disproportionate demand (H6b), and illegitimate complaint (H6c) on employees’ surface acting are weaker (stronger) when customer orientation is high (low).

*H7*: The negative influences of verbal abuse (H7a), disproportionate demand (H7b), and illegitimate complaint (H7c) on employees’ deep acting are weaker (stronger) when customer orientation is high (low).

## Materials and methods

### Sample and procedure

A large telecommunications company with five call centers in China was selected to carry out the survey for three reasons. First, Call center provides a high-contact service characterized by intense customer-employee interaction, which facilitates investigating emotional labor. Second, DCB is very prevalent in call centers, especially for employees who are in charge of dealing with customers’ complaints (Grandey et al., 2004; Boukisa et al., 2020). Finally, call center is frequently used in service research as a representative context to study DCB (Kang and Gong, 2019). With the help of top managers, all frontline employees were encouraged to participate in our survey. Five trained research assistants collected the data on site during shift meetings. To guarantee the validity of the data, before the survey, research assistants introduce the purpose of this research and emphasize anonymity and confidentiality of their responses. It took about 15 min to fill the questionnaire.

A total of 500 questionnaires were distributed in five call centers with 100 for each. When the questionnaires were completed, respondents sealed the questionnaires in an envelope and returned it to the research assistants by themselves. Of the total of 500 distributed questionnaires, 483 were returned. After eliminating 47 responses incomplete, finally, 436 responses were retained for data analysis, with an effective response rate of 87.2%. The sample consisted of 9.63% males and 90.37% females. Participants were of varying ages (6.65% were no more than 21 years of age; 26.38% were between 21 and 25 years of age; 30.28% were between 26 and 30 years of age; 23.39% were between 31 and 35 years of age; 13.30% were more than 35 years of age). Regarding the education level, 21.79% of the participants had a high school education, 78.21% had received college education or above in which 19.72% were bachelor’s degree holders or above.

### Measure

Dysfunctional customer behavior. Dysfunctional customer behavior was measured with the scale developed by Kang and Gong (2019). This scale has been previously adopted in service context. Four items measure verbal abuse (Cronbach’s alpha = 0.885), four items measure disproportionate demand (Cronbach’s alpha = 0.840), and five items measure illegitimate complaint (Cronbach’s alpha = 0.913). Sample items of three subscales include “Customers yelled at me,” “Customers demanded special treatment,” and “Customers complained without reason,” respectively.

Emotional labor. The emotional labor of frontline employees was measured with six items adapted from Brotheridge and Lee (2003). Scale items captured two components (three items for surface acting and three items for deep acting). Sample items include “Hide my true feelings about a situation” and “Make an effort to actually feel the emotions that I need to display to others.” The Cronbach’s alphas for surface acting and deep acting scales were 0.900 and 0.899, respectively.

Perceived organizational support. Perceived organizational support was measured by the six-item scale developed by Eisenberger et al. (1997) (Cronbach’s alpha = 0.870). A sample item was “My organization cares about my opinions “.

Customer orientation. The five-item scale developed by Susskind et al. (2003) was used to measure customer orientation in our study (Cronbach’s alpha = 0.866). Sample items include “If possible, I meet all requests made by my customers.” In addition, for all measures, without demographical variables, a five-point Likert-type scale was used ranging from 1 to 5 with 1 indicating “strongly disagree” and 5 indicating “strongly agree.”

Control variables. To reduce the likelihood of employees’ demographic characteristics confounding the relationships examined, we controlled gender (1 = male; 2 = female), age, tenure, and education. Regarding gender differences, the emotional literature suggests that women show greater emotional expressiveness and exhibit more intensive emotional labor during service interaction than men (Cetin et al., 2018). We controlled age and tenure, as prior research suggests that older employees are likely to report higher self-control of emotional expressions during service interactions (Hyun Jung, 2016). Rasheed-Karim (2020) also reported that age affects the frequency of emotional labor.

### Common method bias, confirmatory factor analysis, and reliability

To rule out the possibility of common method bias, we conducted Harman’s one-factor test to examine the common method variance (CMV; Podsakoff and Organ, 1986). Seven factors with eigenvalues exceeding 1 were extracted from factor analysis. The variance explained for the first factor accounted for only 24.85% of the total variance. Therefore, CMV was not a problem in this study.

Confirmatory factor analysis (CFA) was conducted to assess the convergent and discriminant validity of all of the constructs. CFA is widely used for validity test (Bentler and Bonett, 1980; Ahmed et al., 2021). We used one measurement model to estimate all the focal latent constructs (verbal abuse, disproportionate demand, illegitimate complaint, surface acting, deep acting, perceived organizational support, and customer orientation). The model fit indices were used in this study: normed fit index (NFI > 0.90), comparative fit index (CFI > 0.90), incremental fit index (IFI > 0.90), goodness of fit index (GFI > 0.90), root mean residual (RMR < 0.05), root mean square error of approximation (RMSEA < 0.05). The overall fit indices for the measurement model indicate an acceptable fit to the data: *χ*2/df = 1.560, *p* < 0.001; NFI = 0.925; CFI = 0.971; IFI = 0.972; GFI = 0.919, RMR = 0.048, RMSEA = 0.036. The factor loadings of all items were greater than 0.6. Next, according to the procedures proposed by Fornell and Larcker (1981), the results showed that the square roots of average variance extracted values (AVE) for all constructs, ranging from 0.729 to 0.869 (see Table 1), were greater than the correlation between all constructs (see Table 1). Therefore, these results indicate that the convergent validity and discriminant validity of all constructs are acceptable. Finally, the reliability of all constructs in our research is satisfactory, with the Cronbach’s alpha ranging from 0.840 to 0.913 (see Table 1). Table 1 also contains the means, standard deviations, and intercorrelations of constructs involved in this study.

**Table 1:** Descriptive analysis, correlations, reliabilities, and tests of discriminant validity for all research variables.

Variables	Means	SD	Correlation Matrix							*α*
			1	2	3	4	5	6	7	
1. Verbal abuse	3.223	0.950	**0.817**							0.885
2. Disproportionate demand	3.129	0.861	0.454^***^	**0.754**						0.840
3. Illegitimate complaint	3.275	0.944	0.620^***^	0.505^***^	**0.824**					0.913
4. Surface acting	3.658	0.893	0.342^***^	0.122^*^	0.340^***^	**0.869**				0.900
5.Deep acting	3.700	0.939	−0.331^***^	−0.334^***^	−0.384^***^	−0.047	**0.867**			0.899
6. Perceived organizational support	3.413	0.897	−0.202^***^	−0.071	−0.069	−0.191^***^	0.216^***^	**0.729**		0.870
7. Customer orientation	3.842	0.938	−0.125^*^	0.091	−0.086	−0.169^**^	0.173^**^	0.284^***^	**0.757**	0.866

The square roots of AVE are presented in diagonal elements (bold values).

* *p* < 0.05;

** *p* < 0.01;

*** *p* < 0.001.

## Results

### Test of main effects

The study applied the hierarchical regression analysis to test the hypotheses, and the results are detailed below and summarized in Table 2. Specifically, we use the SPSS 22.0 to process and analyzed data. Hypothesis 1 to 3 predicted the relationship between the three types of DCB (verbal abuse, disproportionate demand, and illegitimate complaint) and employees’ emotional labor strategies.

**Table 2:** Test of main effect.

Independent variable		Surface acting		Deep acting	
		M1	M2	M-3	M-4
Control variable	Gender	−0.131^***^	−0.095^*^	−0.067	−0.078
	Age	0.072	0.072	−0.136^*^	−0.148^**^
	Education	−0.009	0.007	0.003	0.000
	Tenure	−0.063	−0.116^*^	−0.068	−0.001
Verbal abuse			0.230^***^		−0.144^**^
Disproportionate demand			−0.061		−0.140^**^
Illegitimate complaint			0.217^***^		−0.184^**^
*R* ^2^		0.020	0.153	0.038	0.179
*F*		2.225	11.084^***^	4.283^**^	13.305^***^

* *p* < 0.05;

** *p* < 0.01;

*** *p* < 0.001.

To test the effects of DCB on surface acting, we firstly regressed surface acting on control variables (M-1) and then on verbal abuse, disproportionate demand, and illegitimate complaint (M-2). As shown in Table 2, both customers’ verbal abuse (*β* = 0.230, *p* < 0.001) and illegitimate complaint (*β* = 0.217, *p* < 0.001) are positively related to employees’ surface acting, supporting H1a and H3a. However, disproportionate demand was not significantly related to surface acting (M-2 in Table 2, *β* = −0.061, *p* > 0.05), and H2a was rejected.

To test the effects of DCB on deep acting, we regressed deep acting on verbal abuse, disproportionate demand, and illegitimate complaint (M-4). As shown in Table 2, H1b, H2b, and H3b were supported because the effects of customers’ verbal abuse (*β* = −0.144, *p* < 0.01), disproportionate demand (β = −0.140, p < 0.01), and illegitimate complaint (*β* = −0.184, *p* < 0.01) on employees’ deep acting were both significant and negative.

### Test of moderation effects of POS and CO

To test the moderating effects of POS and customer orientation, we conducted a series of hierarchical regression analyses. In line with Aiken et al. (1991) guidelines for moderated regression, we mean-centered the independent variables (verbal abuse, disproportionate demand, and illegitimate complaint) and moderators (POS and customer orientation) at their own mean before creating interaction terms. The variables were entered sequentially in the regression model: First the main effects, then the interaction items.

To test the moderating effects of POS, as we predicted in H4 and H5, a series of moderated regressions were conducted (M-5 to M-12). The results are presented in Table 3. Specially, we first test the main effects as shown in M-5 in Table 3. The results indicate that verbal abuse (*β* = 0.208, *p* < 0.001) and illegitimate complaint (*β* = 0.227, p < 0.001) are positively related to surface acting, while POS is negatively related to surface acting (β = −0.116, *p* < 0.05). Then, we entered the verbal abuse × POS interaction term in M-6 and its effect on surface acting is negative and significant (*β* = −0.265, *p* < 0.001). In addition, the changes in *R*-squared between M-5 and M-6 are significant (△*R*^2^ = 0.064, *p* < 0.001), thus POS significantly moderates the relationship between verbal abuse and surface acting, supporting H4a. However, the disproportionate demand × POS interaction term’s effect on surface acting is not significant (M-7) and H4b is rejected. According to the results of M-8, illegitimate complaint × POS is significantly related to surface acting (*β* = −0.132, *p* < 0.01), and the changes in *R*-squared between M-5 and M-8 are also significant (△*R*^2^ = 0.016, *p* < 0.01), supporting H4c.

**Table 3:** The moderation effect of Perceived organizational support.

Independent variable		Surface acting				Deep acting			
		M-5	M-6	M-7	M-8	M-9	M-10	M-11	M-12
Control variable	Gender	−0.075	−0.090^*^	−0.076	−0.095^*^	−0.106^*^	−0.103^*^	−0.098^*^	−0.098^*^
	Age	0.060	0.054	0.059	0.053	−0.131^**^	−0.129^**^	−0.116^*^	−0.127^**^
	Education	0.016	0.017	0.015	0.009	−0.013	−0.014	−0.009	−0.011
	Tenure	−0.119^*^	−0.123^*^	−0.119^*^	−0.116^*^	0.004	0.005	0.005	0.003
Verbal abuse		0.208^***^	0.146^**^	0.207^***^	0.201^***^	−0.111^*^	−0.098	−0.109^*^	−0.108
Disproportionate demand		−0.065	−0.042	−0.066	−0.049	−0.133^**^	−0.138^**^	−0.125^*^	−0.140^**^
Illegitimate complaint		0.227^***^	0.226^***^	0.228^***^	0.202^***^	−0.199^***^	−0.198^***^	−0.205^***^	−0.188^**^
POS		−0.116^*^	−0.076	−0.115^*^	−0.103^*^	0.174^***^	0.166^***^	0.163^***^	0.169^***^
Verbal abuse × POS			−0.265^***^				0.057		
Disproportionate demand × POS				−0.015				0.120^**^	
Illegitimate complaint × POS					−0.132^**^				0.056
R^2^		0.166	0.230	0.166	0.182	0.207	0.210	0.221	0.210
F		10.627^***^	14.177^***^	9.439^***^	10.551^***^	13.926^***^	12.575^***^	13.415^***^	12.573^***^
△R^2^			0.064^***^	0.000	0.016^**^		0.003	0.014^**^	0.003

* *p* < 0.05;

** *p* < 0.01;

*** *p* < 0.001.

The results of M-9 to M-12 indicated moderating effects of POS on the relationships between DCB and deep acting. Specifically, the results of Model 9 (M-9) indicated that both verbal abuse (*β* = −0.111, *p* < 0.05), disproportionate demand (*β* = −0.133, *p* < 0.01), and illegitimate complaint (*β* = −0.199, *p* < 0.001) were negatively related to deep acting, while POS was positively related to deep acting (*β* = 0.174, *p* < 0.001). The interactions term of POS and verbal abuse did not affect deep acting significantly and H5a was rejected (M-10). When the interaction item of POS and disproportionate demand was entered in M-11, the results indicated that the interaction term was significantly related to deep acting (*β* = 0.120, *p* < 0.01). The changes in R-squared between M-9 and M-11 were significant (△*R*^2^ = 0.014, *p* < 0.01). H5b was supported. Finally, the interaction item of POS and illegitimate complaint did not affect deep acting significantly and H5c was rejected.

**Figure 2:**
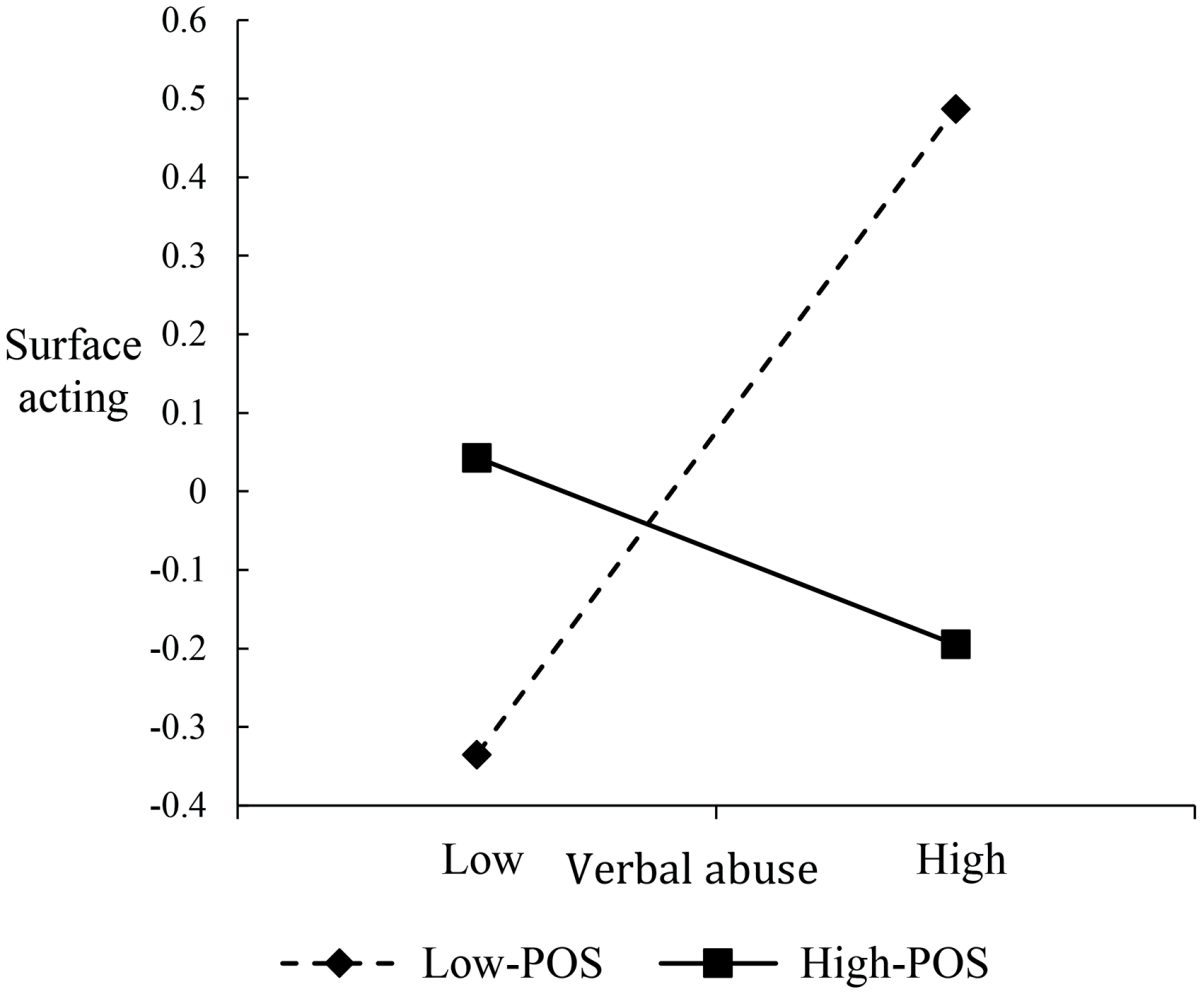
Moderating effect of POS on the relationship between verbal abuse and surface acting.

**Figure 3:**
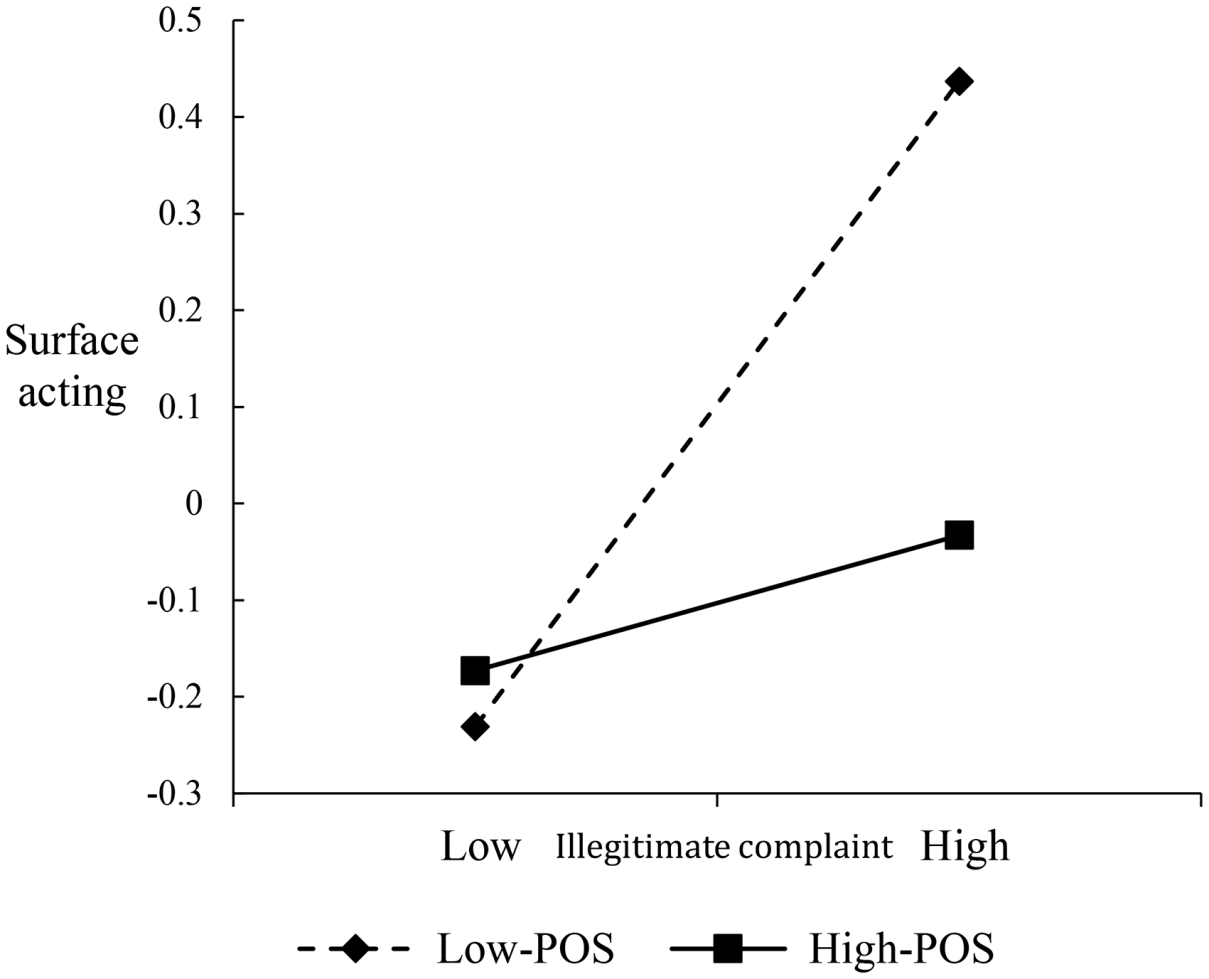
Moderating effect of POS on the relationship between illegitimate complaint and surface acting.

**Figure 4:**
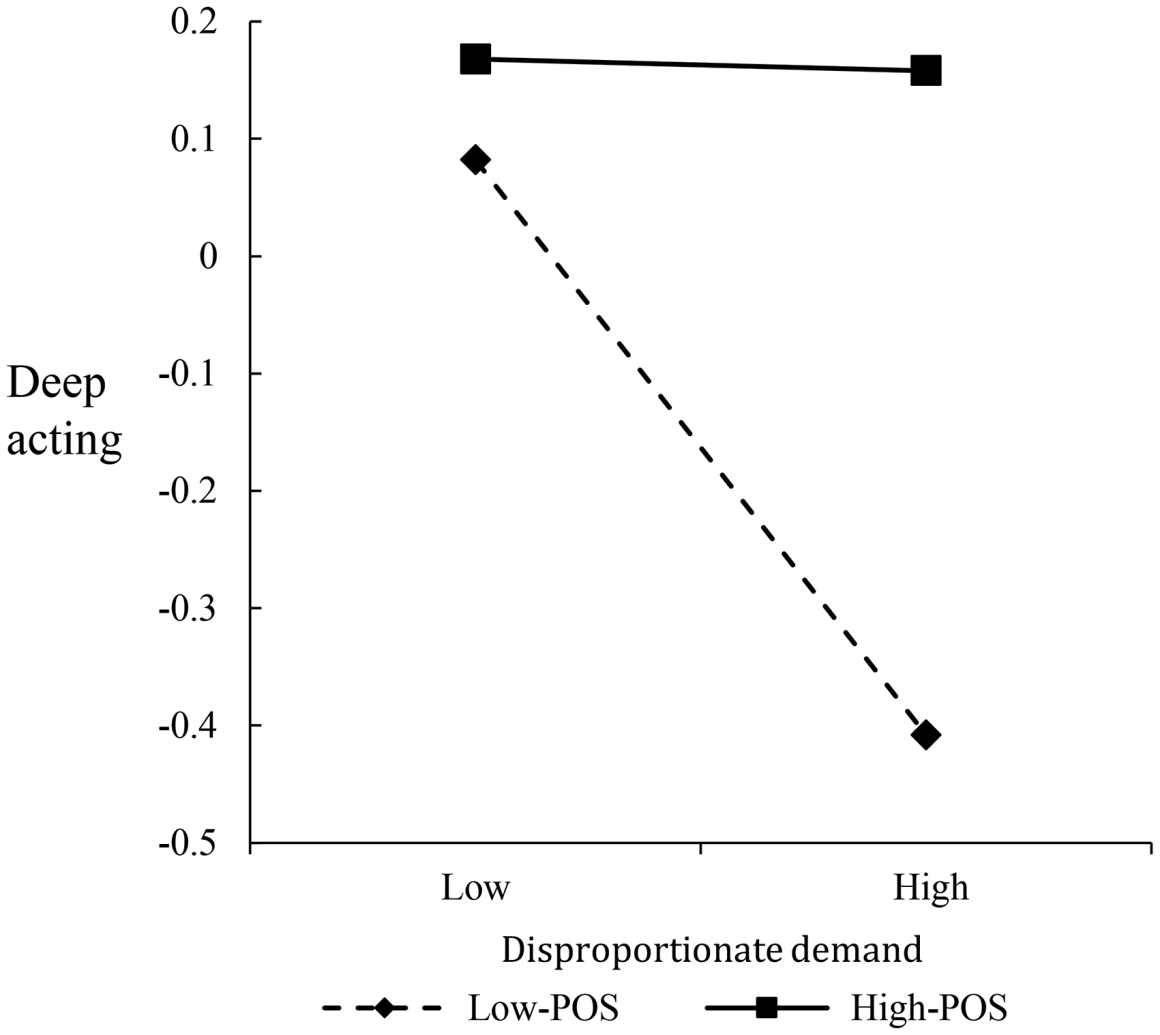
The moderating effect of POS on disproportionate demand—deep acting.

To interpret the nature of the interactions, we plotted the verbal abuse-surface acting, illegitimate complaint-surface acting, and disproportionate demand-deep acting relationships at different levels of POS (i.e., 1 SD above/below the mean), respectively (Figures 2–4). Both verbal abuse-surface acting and illegitimate complaint-surface acting relationships were more strongly positive among frontline employees with low POS than among frontline employees with high POS (Figures 2, 3). For the disproportionate demand-deep acting relationship, when frontline employees reported high POS, the negative relationship is weakened (Figure 4).

To test the moderating effect of CO, as predicted in H6 and H7, we ran a series of moderated regressions (M-13 to M-20). The results were presented in Table 4. Especially, the results of main effects test (M-13) showed that CO was negatively related to surface acting (*β* = −0.113, *p* < 0.05). Then, the interaction term of CO and verbal abuse was entered in M-14. The results suggested that the interaction term was negatively related to surface acting (*β* = −0.173, *p* < 0.001), and changes in *R*-squared between M-13 and M-14 were significant (△*R*^2^ = 0.027, *p* < 0.001), supporting H6a. However, neither interaction item of CO and disproportionate demand nor the interaction item of CO and illegitimate complaint affects surface acting significantly (M-15 and M-16). Thus, hypotheses 6b and 6c were rejected.

**Table 4:** The moderation effect of customer orientation.

Independent variable		Surface acting				Deep acting			
		M-13	M-14	M-15	M-16	M-17	M-18	M-19	M-20
Control variable	Gender	−0.077	−0.071	−0.062	−0.082	−0.104^*^	−0.105^*^	−0.041	−0.095^*^
	Age	0.074	0.060	0.075	0.070	−0.151^**^	−0.147^**^	−0.144^**^	−0.143^**^
	Education	0.010	0.022	0.011	0.014	−0.005	−0.008	0.002	−0.014
	Tenure	−0.116^*^	−0.126^*^	−0.113^*^	−0.117^*^	−0.001	0.002	0.013	0.002
Verbal abuse		0.218^***^	0.222^***^	0.215^***^	0.229^***^	−0.127^*^	−0.128^*^	−0.138^**^	−0.148^**^
Disproportionate demand		−0.044	−0.065	−0.034	−0.058	−0.165^**^	−0.159^**^	−0.125^*^	−0.138^**^
Illegitimate complaint		0.211^***^	0.223^***^	0.215^***^	0.204^***^	−0.174^**^	−0.177^**^	−0.157^**^	−0.162^**^
CO		−0.113*	−0.062	−0.115^*^	−0.092	0.171^***^	0.157^**^	0.164^***^	0.129^**^
Verbal abuse × CO			−0.173^***^				0.047		
Disproportionate demand × CO				0.059				0.238^***^	
Illegitimate complaint × CO					−0.086				0.165^***^
*R* ^2^		0.166	0.192	0.169	0.172	0.206	0.208	0.256	0.231
*F*		10.594^***^	11.276^***^	9.603^***^	9.855^***^	13.874^***^	12.450^***^	16.296^***^	14.222^***^
△*R*^2^			0.027^***^	0.003	0.007		0.002	0.050^***^	0.025^***^

* *p* < 0.05;

** *p* < 0.01;

*** *p* < 0.001.

Hypothesis 7 predicted that CO moderates the effects of customer verbal abuse (H7a), disproportionate demand (H7b), and illegitimate complaint (H7c) on employees’ deep acting. The results of the main effect test, as shown in M-17, suggested that CO was positively related to deep acting (*β* = 0.171, *p* < 0.001). The interaction term of CO and verbal abuse was not significantly related to deep acting (M-18). H7a was rejected. In M-19, the interaction term of CO and disproportionate demand was positively related to deep acting (*β* = 0.238, *p* < 0.001). The changes in R-squared between M-17 and M-19 were significant (△*R*^2^ = 0.050, *p* < 0.001), supporting H7b. Finally, in M-20, the interaction item of CO and illegitimate complaints was positively related to deep acting (*β* = 0.165, *p* < 0.001), and changes in *R*-squared between M-17 and M-20 were also significant (△*R*^2^ = 0.025, *p* < 0.001), supporting H7c.

**Figure 5:**
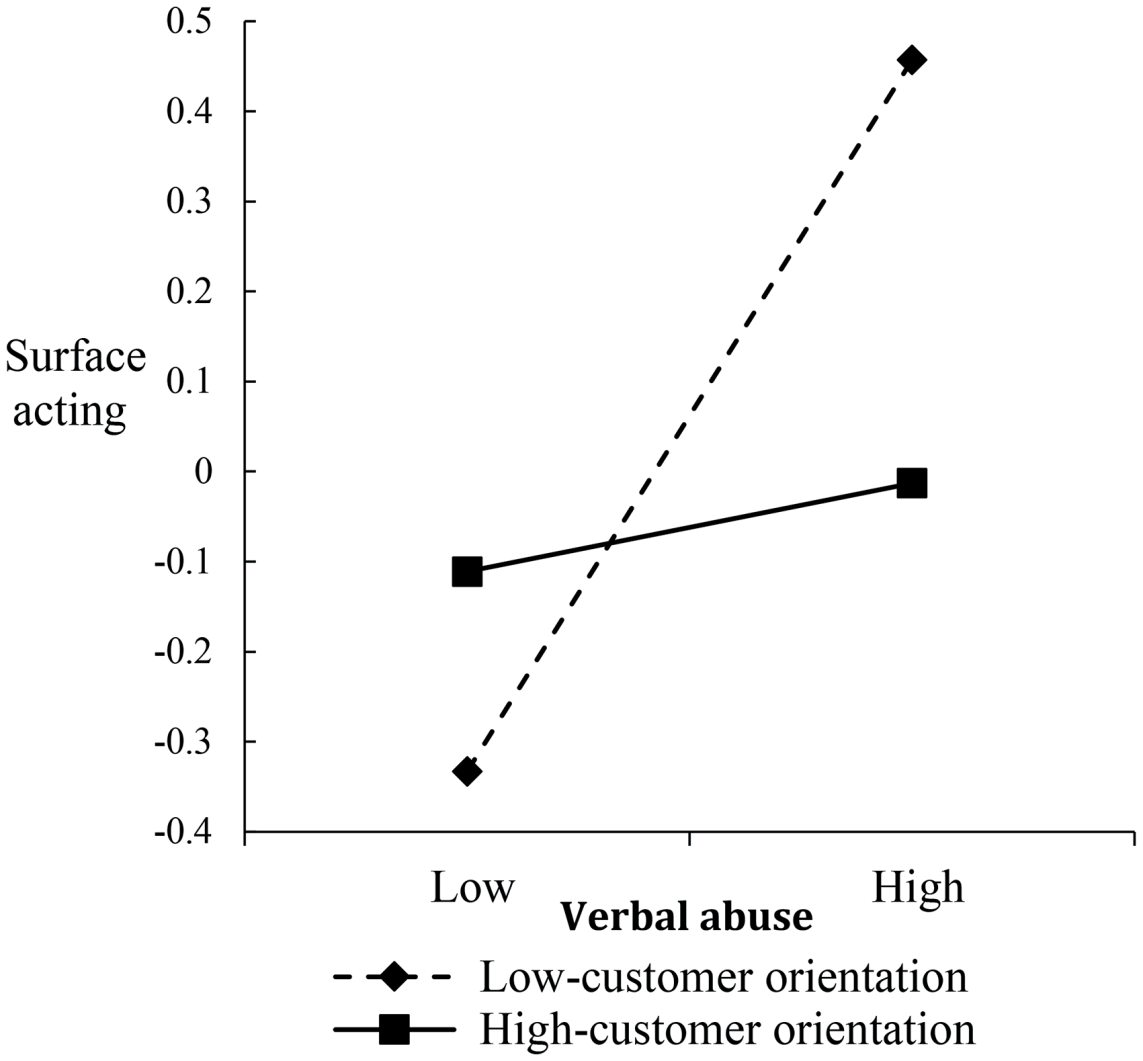
Moderating effect of customer orientation on the relationship between verbal abuse and surface acting.

**Figure 6:**
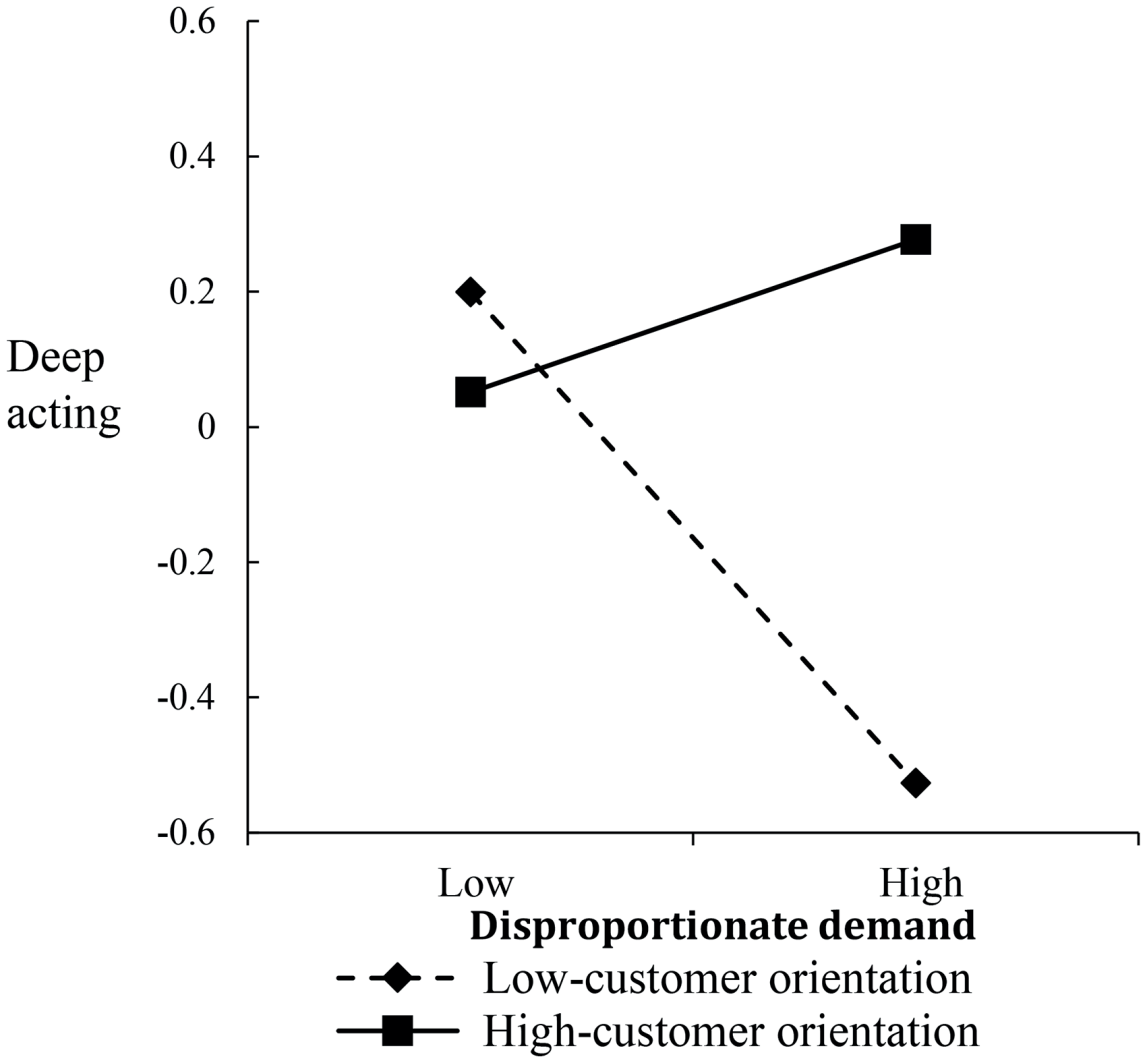
Moderating effect of customer orientation on the relationship between disproportionate demand and deep acting.

**Figure 7:**
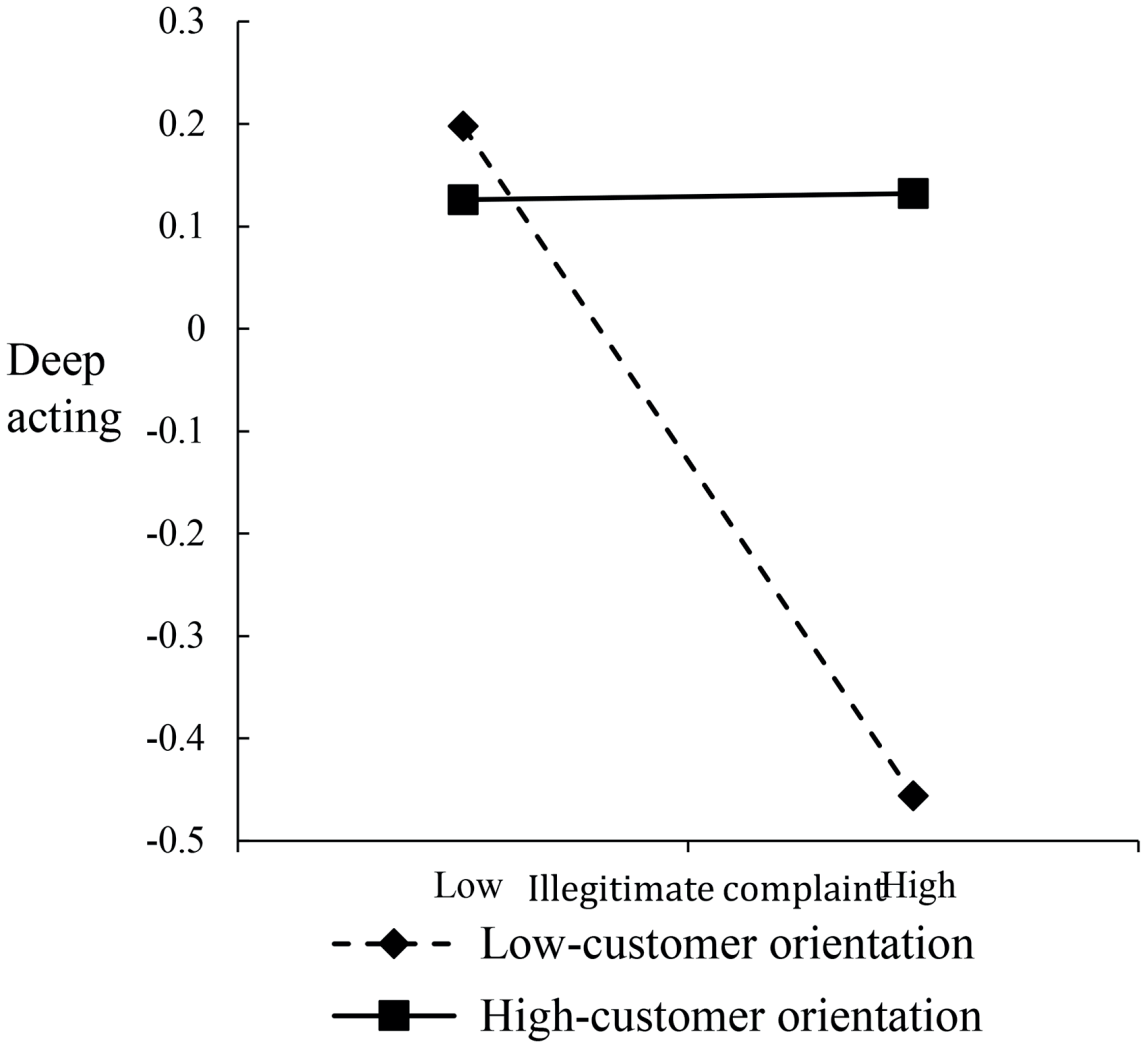
Moderating effect of customer orientation on the relationship between illegitimate complaint and deep acting.

To interpret the nature of the interactions, we plotted the verbal abuse-surface acting, disproportionate demand-deep acting, and illegitimate complaint-deep acting relationships at different levels of CO (i.e., 1 SD above/below the mean), respectively (Fifure5–7). For the verbal abuse-surface acting relationship, when frontline employees reported high CO, the postive relationship was weakened (Figure 5). Both disproportionate demand-deep acting and illegitimate complaint-deep acting relationships were more strongly negative among frontline employees with low CO than among frontline employees with high CO (Figures 6, 7).

## Discussion and conclusion

As the prevalence of DCB in many service sectors, how to cope with this challenge becomes an interesting question that attracts the attention of both researchers and practitioners (Szczygiel and Bazińska, 2021; Zhan et al., 2021). We developed and tested a conceptual model of the differential influence of the three types of DCB on employees’ emotional labor strategies (surface acting and deep acting). Also, the moderating effects of POS and customer orientation on these relationships were examined. Using data from 436 frontline employees in call centers, the results demonstrate that customers’ verbal abuse and illegitimate complaint directly and positively affect employee surface acting, while verbal abuse, disproportionate demand, and illegitimate complaint directly and negatively impact employees’ deep acting. POS negatively moderates the effects of verbal abuse and illegitimate complaint on surface acting, while positively moderates the influence of disproportionate demand on deep acting. We also found that the positive effect of verbal abuse on surface acting and the negative effect of disproportionate demand and illegitimate complaints on deep acting are weaker in conditions of high (rather than low) customer orientation.

### Theoretical contributions

This research contributes to the literature on dysfunctional customer behavior and emotional labor. First, our study extends previous research on DCB by enriching the knowledge on the consequences of DCB. Drawing on COR theory, we proposed and empirically tested the relationships between three types of DCB on emotion labor. Harris and Reynolds (2003) proposed three categories of consequences of DCB: consequences for customer contact employees, consequences for customers, and consequences for organizations. Although not be empirically tested, their propositions and callings for exploring consequences of DCB more holistically have inspired many scholars to seek consequences of DCB mainly from employee, customer, and organization perspectives. Different from prior research focusing on the “long-term” effects of DCB on employees’ wellbeing (such as burnout, emotional exhaustion, job anxiety, and job stress; Bamfo et al., 2018; Gong and Wang, 2019; Raza et al., 2021; Szczygiel and Bazińska, 2021), our study aims to explore how frontline employees’ response DCB instantly during the service encounters. By empirically examining DCB’s effect on frontline employees’ encounter-specific behavior—emotional labor, our study answers Harris and Reynolds’s (2003) calls and fills the gap of DCB’s instant effects. Furthermore, different from most of the previous research focusing on one specific form of DCB (Grandey et al., 2004; Park and Kim, 2019; Gong and Wang, 2021), our study, consist with Kang and Gong (2019), tests the effects of three types of employee-targeting DCB on emotional labor simultaneously. By doing so, we can compare the relative strength of different types of DCB’s effects on emotional labor. For instance, both verbal abuse and illegitimate complaint are positively related to surface acting, while disproportionate demand is not.

Second, we also advance research on emotional labor from the perspective of customer. Given the interactive nature of service encounters and the importance of employees’ emotional labor (Ashtar et al., 2021), identifying the antecedents of emotional labor from customer perspective is warranted. Previous research has indicated that customer misbehaviors were positively related to employee emotional labor (Sliter et al., 2010; Hu and King, 2017). Customer incivility was found to be associated with employees’ surface acting as well (Hur et al., 2015; Szczygiel and Bazińska, 2021). However, few studies examined the relationship between DCB and different emotional labor strategies, particularly the effect of DCB on employees’ deep acting. To our knowledge, our study is the first to empirically elucidate the relationships between these three different types of dysfunctional customer behaviors and two emotional labor strategies among call centers’ frontline employees, which helps to enrich the literature on the antecedents of emotional labor.

Third, what had received little attention to the boundary conditions under which DCB exerts more or less detrimental effects on employee emotional labor. Perceived organizational support has been shown to moderate the effects of DCB on employees’ wellbeing and behaviors, such as burnout (Han et al., 2016), employee service sabotage (Hwang et al., 2021), and job anxiety (Raza et al., 2021). Our study demonstrated that POS (as external resources) and customer orientation (as internal resources) attenuate the effects of different types of DCB on emotional labor of frontline employees, provided a better understanding of the mechanism underlying the influence, and contributed to the previous literature. Specifically, our findings suggest that frontline employees with high POS are likely to engage in deep acting when they are confronted with disproportionate demand. Similarly, supervisor support has been shown to moderate the influence of interpersonal mistreatment on nurses’ deep acting (Goussinsky and Livne, 2016). Furthermore, when frontline employees with high POS suffer from customer verbal abuse and illegitimate complaint, they are not even willing to pretend positive emotions (surface acting). This finding is consistent with the latest research by Huang et al. (2021). They argue that necessary evil (e. g. displaying negative emotions), refers to action that can cause unpleasant experiences to dysfunctional customers, is a new effective way for frontline employees to cope with DCB. In addition, although we highlight that DCB is associated with frontline employees’ emotional labor, customer orientation helps alleviate this relationship, which provides a new prospect for further exploration.

### Managerial implications

Our findings shed light on how service firms can enhance frontline employees’ ability to cope with DCB. First, given the important role of frontline employees’ emotional labor in creating good service experience, service firms should improve frontline employees’ emotional skills by setting training plans. For example, managers can design scripts and scenarios to simulate DCB context and help employees to rebuild service processes and routines to cope with DCB more effectively. Once the new process and routines are formed, frontline employees could cope with DCB at low cost of resource depletion. That means employees can invest more cognitive efforts to engage in deep acting, rather than surface acting.

Second, considering the harmful consequences of DCB, service firms should monitor the level of DCB. At the end of the workday, managers could organize a short conversation with frontline employees and talk about DCB they have just suffered. If the service firm is equipped with surveillance system, DCB can also be monitored by analyzing the recording data.

Finally, considering the buffer effects of POS and customer orientation, we recommend managers improve the level of frontline employees’ customer orientation by recruiting employees who are customer-oriented. Managers can screen applicants by setting a survey that contains customer orientation scales before the interview. Also, service firms can improve employees’ customer orientation by cultivating service climate. To improve POS, managers, as representatives of organization, should take measures to support their employees. Specifically, managers can take empowering leadership which can enhance employees’ perception of respect and trust in the organization. Service firms should carefully create a trusting and supportive climate and make frontline employees feel support from their organization.

### Limitations and future directions

Some limitations should be noted. First, the sample in this study was collected at the call centers. While call centers fit into the context of the current research, which may limit the generalizability of our findings to other industries. Future research may consider choosing other industries (e.g., hotels). Second, due to the specific characteristics of service encounters in call centers, this study examined the relationship between DCB and employees’ emotional labor when only one customer was present. To enhance generalizability of the findings, future studies should examine whether employees respond to DCB in the same way when other customers are present. Third, this study merely examined the moderating roles of POS and customer orientation. Future studies should consider other organizational factors’ moderating effect on the relationship between DCB and emotional labor such as organizational culture which comprises basic internalized beliefs and values that guide employees’ perceptions and actions. Furthermore, the present research focused on the effects of DCB on employees’ emotional labor, while downstream consequences of this mechanism might also be meaningful such as burnout. Finally, this study tested the hypotheses using data collected from employee perspective. Although we did take some steps to reduce the influence of common method bias such as randomizing the order of measures, future research could collect employee-customer dyadic data or use multiple time points to reduce the possible common method bias and self-reporting bias.

## Data availability statement

The raw data supporting the conclusions of this article will be made available by the authors, without undue reservation.

## Author contributions

PC and SX contributed to the original idea and designed the study. JJ performed the statistical analysis and wrote the first draft of the manuscript. PC, JJ, and ZL revised it critically for important intellectual content. All authors contributed to the article and approved the submitted version.

## Funding

This work was funded by the Soft Science Research Program of Shaanxi Province of China (NO: 2021KRM157) and Scientific Research Program Funded by Shaanxi Provincial Education Department (Program No. 21JZ041).

## Conflict of interest

The authors declare that the research was conducted in the absence of any commercial or financial relationships that could be construed as a potential conflict of interest.

## Publisher’s note

All claims expressed in this article are solely those of the authors and do not necessarily represent those of their affiliated organizations, or those of the publisher, the editors and the reviewers. Any product that may be evaluated in this article, or claim that may be made by its manufacturer, is not guaranteed or endorsed by the publisher.
